# Preoperative ultrasound identification and localization of the inferior parathyroid glands in thyroid surgery

**DOI:** 10.3389/fendo.2023.1094379

**Published:** 2023-02-27

**Authors:** Ruyue Chen, Kaining Zhang, Ju Liu, Ling Guo, Kailin Liu, Chong Geng

**Affiliations:** ^1^ Department of Breast and Thyroid Surgery, Shandong Provincial Hospital Affiliated to Shandong First Medical University, Jinan, Shandong, China; ^2^ Department of Ultrasonography, Shandong Provincial Hospital Affiliated to Shandong First Medical University, Jinan, Shandong, China; ^3^ Department of Anesthesiology, Shandong Provincial Hospital Affiliated to Shandong First Medical University, Jinan, Shandong, China

**Keywords:** thyroid cancer, parathyroid gland, hypoparathyroidism, ultrasound localization, clinical effect

## Abstract

**Introduction:**

The parathyroid glands are important endocrine glands for maintaining calcium and phosphorus metabolism, and they are vulnerable to accidental injuries during thyroid cancer surgery. The aim of this retrospective study was to investigate the application of high-frequency ultrasound imaging for preoperative anatomical localization of the parathyroid glands in patients with thyroid cancer and to analyze the protective effect of this technique on the parathyroid glands and its effect on reducing postoperative complications.

**Materials and methods:**

A total of 165 patients who were operated for thyroid cancer in our hospital were included. The patients were assigned into two groups according to the time period of surgery: Control group, May 2018 to February 2021 (before the application of ultrasound localization of parathyroid in our hospital); PUS group, March 2021 to May 2022. In PUS group, preoperative ultrasound was used to determine the size and location of bilateral inferior parathyroid glands to help surgeons identify and protect the parathyroid glands during operation. We compared the preoperative ultrasound results with the intraoperative observations. Preoperative and first day postoperative serum calcium and PTH were measured in both groups.

**Results:**

Our preoperative parathyroid ultrasound identification technique has more than 90% accuracy (true positive rate) to confirm the location of parathyroid gland compared to intraoperative observations. Postoperative biochemical results showed a better Ca^2+^ [2.12(0.17) vs. 2.05(0.31), P=0.03] and PTH [27.48(14.88) vs. 23.27(16.58), P=0.005] levels at first day post-operation in PUS group compared to control group. We also found a reduced risk of at least one type of hypoparathyroidism after surgery in control group:26 cases (31.0%) vs. 41 cases (50.6%), p=0.016.

**Conclusion:**

Ultrasound localization of the parathyroid glands can help in the localization, identification and *in situ* preservation of the parathyroid glands during thyroidectomy. It can effectively reduce the risk of hypoparathyroidism after thyroid surgery.

## 1 Introduction

Thyroid cancer is one of the most rapidly increasing malignancies in the world in recent years, and more than 90% of its pathological type is papillary carcinoma, for which surgical resection represents the most effective treatment (1). Hypoparathyroidism caused by surgical damage is one of the most common comorbidities of thyroid surgery (2, 3).

The parathyroid gland (PG) is an important endocrine gland. The chief cells of PG secrete parathyroid hormone (PTH), an important hormone that maintaining calcium and phosphorus homeostasis in the body (4, 5). Due to different migration patterns during embryonic development, Inferior parathyroid glands (IPG) ectopia is observed frequently with a fragile blood supply and anatomical variability (6, 7). These locations include paraoesophageal, mediastinal, intrathoracic, intrathyroidal and/or around the carotid sheath (8). Additionally, the number of PGs is also varying (9). Therefore, it is crucial to identify and protect the PGs *in situ* during thyroidectomy (10, 11), because incidental resection of one or more PGs or disruption of parathyroid blood supply may lead to hypocalcemia and hypoparathyroidism (12, 13), causing symptoms such as numbness sensation in the hands and feet and even paralysis of the laryngeal and respiratory muscles, which seriously affects the quality of life of patients. It is also a major cause of doctor-patient tension after thyroid surgery.

Ultrasound diagnostic techniques is a common examination of thyroid and cervical lymph node lesions (14, 15). Compared to other diagnostic techniques, ultrasound diagnosis has several advantages including dynamic, radiation-free, reproducible, high-resolution, low-cost, and is accepted by a larger number of patients (16, 17). However, studies on the characteristics of preoperative normal parathyroid ultrasound images are not very prominent (18, 19), and it is desirable to improve the clinical knowledge and application of preoperative normal parathyroid ultrasound images.

The purpose of this work is to evaluate the protective effect of the application of preoperative high-frequency ultrasound imaging of parathyroid in patients with thyroid cancer and its effect on reducing postoperative complications.

## 2 Materials and methods

### 2.1 Patience

A total of 165 patients with thyroid cancer surgery in our hospital from May 2018 to May 2022 were included, with a male to female ratio of 38:127 (1:3.34), aged from 21 to 71 years, with a mean of 45.63 ± 11.29 years.

The patients were assigned into two groups according to the time period of surgery: Control group, May 2018 to February 2021 (before the application of ultrasound localization of parathyroid in our hospital); PUS group, March 2021 to May 2022 (After the application of ultrasound localization of parathyroid). Among them, 81 cases were in the control group and 84 cases in the preoperative ultrasound group (PUS group).

Inclusion criteria: (1) Subject is received total thyroidectomy and bilateral central node dissection, and is diagnosed as papillary thyroid cancer by postoperative pathology. (2) Subject is treated for the first time with thyroid surgery. (3) Preoperative parathyroid hormone levels, serum calcium levels, liver function and kidney function were within normal range. No history of suspected parathyroid pathology such as chronic kidney disease, urinary tract stones, increased or decreased bone density, pathological fracture, etc.

Exclusion criteria: (1) Subjects is received lymph node dissection in the lateral neck area. (2) Subjects is received intraoperative parathyroid auto-transplantation.

All subjects were informed and the informed consent form were signed. The study was approved by the ethics committee of our hospital (NO.2022-301).

### 2.2 Identification of normal Inferior parathyroid glands using ultrasonography

**Figure 1:**
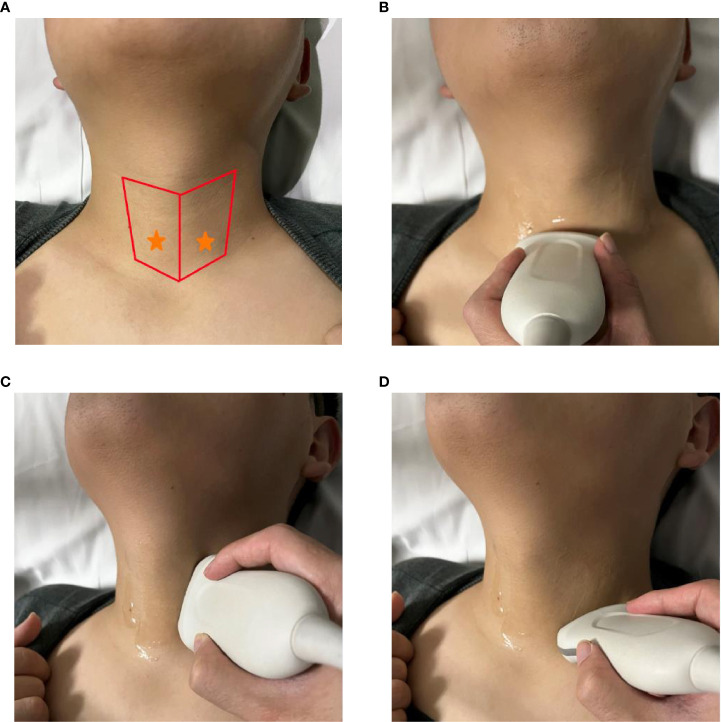
Identification of normal Inferior parathyroid glands using ultrasonography. **(A)** red area: main scanning range, yellow star: expected location of IPGs; **(B–D)** operation method of scanning IPGs.

**Figure 2:**
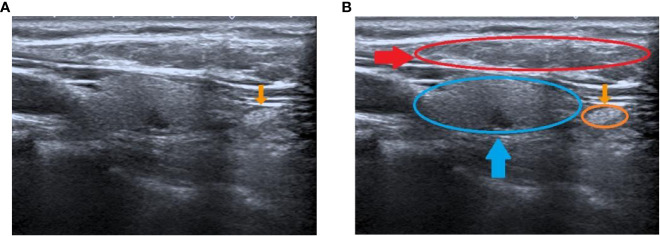
Preoperative ultrasound identification of the IPG. **(A)** yellow arrow shows the IPG; **(B)** yellow arrow and circle show the IPG, blue arrow and circle show the thyroid, red arrow and circle show the muscle.

We developed a ultrasonic identification method of the IPGs. The IPGs was scaned using Aplio 500 Doppler ultrasonography (Toshiba) with a 5 to 14 MHz frequency probe. During the examination, the patient was placed in a supine position, and the head was tilted back to fully expose the anterior neck, with the head tilted to the side if necessary (**Figure 1**). The number, location, size, and internal echogenicity of the IPGs are observed (**Figure 2**).

Operation of ultrasonography: The upper and lower range is from the middle and lower 1/2 of the dorsal thyroid to the level of the sternoclavicular joint, and the right and left range is between the trachea and the lateral border of the sternocleidomastoid (**Figure 1**).

Ultrasound features of the IPGs we developed are: (1) The morphology is mostly oval, a few narrow. (2) The size is about (6-8 mm) x (4-5 mm) x (3-4 mm). (3) The overall echogenicity is higher than that of the surrounding muscle tissue and is slightly higher than that of the thyroid tissue. The internal echogenicity is homogenous and a border with the surrounding tissue is visible (**Figure 2**). We have grouped the location of parathyroid into two categories, type I: closely opposed to the thyroid peritoneum; type II: free below the inferior pole of the thyroid gland or lateral to the thyroid gland, not closely opposed to the thyroid gland.

**Figure 3:**
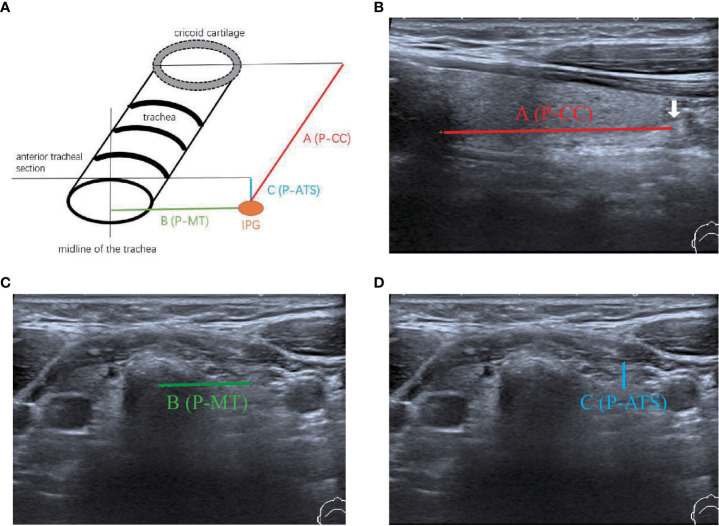
Measurement of the relative location of the IPG to the trachea. **(A)** Diagram of IPG location; **(B)** The distance of the IPG from the cricoid cartilage (A, PCC); **(C)** The distance of the IPG from the midline of the trachea (B, P-MT) ; **(D)** The distance of the IPG from the anterior tracheal section (C, P-ATS).

We have focused on measuring the following three distances of the IPGs in cross-section and longitudinal section: (A) The distance of the IPGs from the cricoid cartilage; (B) The distance of the IPGs from the midline of the trachea; (C)The distance of the IPGs from the anterior tracheal section. (It should be noted that this study did not involve bilateral superior parathyroid glands(SPG); please see the Conclusions and Discussion section for details). The diameters of three axes of the IPG were also measured (**Figure 3**). In addition, our IPG ultrasound images were independently reviewed and determined by three senior ultrasound experts individually. We included the PUS group for their agreement to confirm the IPG images.

### 2.3 Operation and measurement

**Figure 4:**
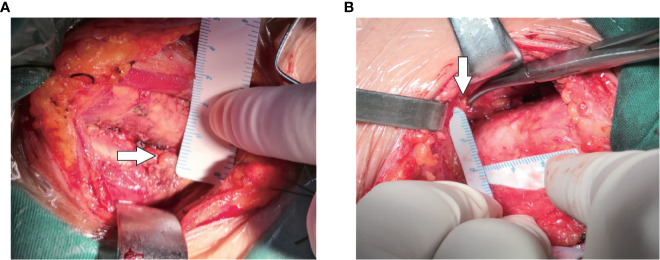
IPG identification and measurement during surgery. **(A, B)** White arrow shows IPG.

Intraoperatively, after carefully searching of the IPGs according the preoperative ultrasound localization, a sterile ruler was used to measure the distance A and B without touching the PGs (**Figure 4**). We didn’t measure the distance C (i.e., the depth), because significant variation due to the pulling of muscles during the anatomical exposure of the thyroid gland. We do not measure the size of the PGs for the sake of unnecessary manipulation.

The Ca^2+^ (SIEMENS, ADVIA2400) and PTH (ROCHE, Cobas e 601) levels were measured preoperatively and on postoperative day 1. Patients with the following conditions are considered to be at risk for hypoparathyroidism: (i) Ca^2+^ less than 2.0 mmol/L; (ii) PTH less than 15 pg/ml; (iii) numbness of mouth and lips; (iv) Tingling and numbness of fingertips; (v) Muscle aches and spasms; (vi) tetany; (vii) laryngeal spasm, diaphragm spasm, and other severe spasms in both skeletal and smooth muscles throughout the body.

### 2.4 Statistical analysis

SPSS 25.0 and Graphpad prism 8.0.2 software were used to process the data. The Mann-Whitney U test was used. Data were expressed as mean ± standard deviation (X ± S) or median (quartiles); Spearman correlation was used for bivariate correlation analysis; Pearson X2 test was used for categorical variables. p<0.05 was considered statistically significant.

## 3 Result

### 3.1 Patient characteristics

A total of 165 eligible patients were included in this study. There were 84 in the preoperative ultrasound group and 81 in the control group. There were no significant differences between the preoperative ultrasound groups and control groups in parameters such as age, sex, prevalence of Hashimoto’s thyroiditis, tumor size, and lymph node metastasis in the central region (**Table 1**).

**Table 1:** Clinical data of patients with thyroid cancer included in this study.

	Total	Control Group	PUS Group	P value
**Age (mean ± SD)**	45.63 ± 11.29	46.48 ± 10.18	44.81 ± 12.27	0.322 †
Gender(n, %)				
Male	38 (23.0%)	18 (22.2%)	20 (23.8%)	0.809 *
Female	127 (77.0%)	63 (77.8%)	64 (76.2%)	
Hashimoto’s thyroiditis(n, %)				
Non-illness	124 (75.2%)	63 (77.8%)	61 (72.6%)	0.443 *
Illness	41 (24.8%)	18 (22.2%)	23 (27.4%)	
Tumor size(n, %)				
<1cm	112 (67.9%)	57 (70.4%)	55 (65.5%)	0.501 *
≥1cm	53 (32.1%)	24 (29.6%)	29 (34.5%)	
Central neck lymph node metastasis(n, %)				
Positive	57 (34.5%)	30 (37.0%)	27 (32.1%)	0.509 *
Negative	108 (65.5%)	51 (63.0%)	57 (67.9%)	

† Mann-Whitney U test.

* Chi-square test.

### 3.2 Preoperative ultrasound localization of IPGs

**Figure 5:**
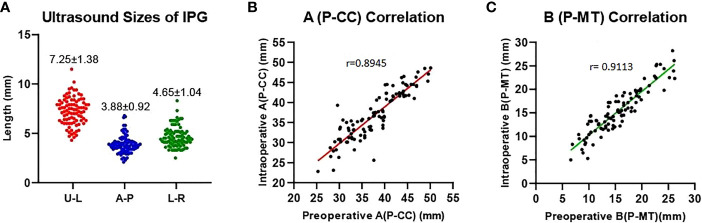
Preoperative ultrasound measurements of the IPG. **(A)** Ultrasound sizes of IPG (U-L: upper-lower diameters, A-P: anterior-posterior diameters, L-R: left-right diameters); **(B, C)** Preoperative and intraoperative distance correlation. Distance of the IPG from the cricoid cartilage (A, P-CC), distance of the IPG from the midline of the trachea (B, P-MT).

Our data showed that the left side IPGs were found in 51 (60.7%) patients and 48 (57.1%) for the right side by ultrasonography. A total of 31 (36.9%) IPGs were found bilaterally, and 68 (81.0%) IPGs were found on at least one side. This indicates that we had a high detection rate of the IPGs on at least one side (**Table 2**). The average size observed in ultrasonography of the IPGs was: (7.25 ± 1.38) x (3.88 ± 0.92) x (4.65 ± 1.04) mm, while the three-dimensional spatial coordinate A, B, and C were (36.70 ± 5.75) mm, (15.85 ± 3.82) mm, and (5.29 ± 2.93) mm, respectively. This is an important reference for finding the IPGs during surgery (**Table 2**; **Figure 5**).

**Table 2:** Details of pre-operative ultrasound evaluation and intra-operative detection of IPG in PUS Group.

	Statistics
Patient numbers pre-operatively (n, %)	
Bilateral IPG detected	31 (36.9%)
At least one IPG detected	68 (81.0%)
Left IPG detected	51 (60.7%)
Right IPG detected	48 (57.1%)
Sizes of IPG pre-operatively (mm, mean ± SD)	
From upper to lower (U-L)	7.25 ± 1.38
From anterior to posterior (A-P)	3.88 ± 0.92
From left to right (L-R)	4.65 ± 1.04
Location distance of IPG pre-operatively (mm, mean ± SD)	
From the cricoid cartilage (P-CC) (A)	36.70 ± 5.75
From the midline of the trachea (P-MT) (B)	15.85 ± 3.82
From the anterior tracheal section (P-ATS) (C)	5.29 ± 2.93
Patient numbers intra-operatively (n, %)	
Bilateral IPG detected	62 (73.8%)
At least one IPG detected	74 (88.1%)
Left IPG detected	70 (83.3%)
Right IPG detected	66 (78.6%)
Location distance of IPG intra-operatively (mm, mean ± SD)	
From the cricoid cartilage (P-CC) (A)	33.70 ± 12.75
From the midline of the trachea (P-MT) (B)	14.79 ± 8.82
Patient numbers comparatively (n, %)	
Left IPG detected both pre- and intra-operatively	41 (48.8%)
Left IPG detected pre-operatively but not intra-operatively	5 (6.0%)
Left IPG detected intra-operatively but not pre-operatively	23 (27.4%)
Right IPG detected both pre- and intra-operatively	45 (53.6%)
Right IPG detected pre-operatively but not intra-operatively	1 (1.2%)
Right IPG detected intra-operatively but not pre-operatively	17 (20.2%)
No IPG detected pre-operatively	16 (19.0%)
No IPG detected both pre- and intra-operatively	6 (7.1%)

**Figure 6:**
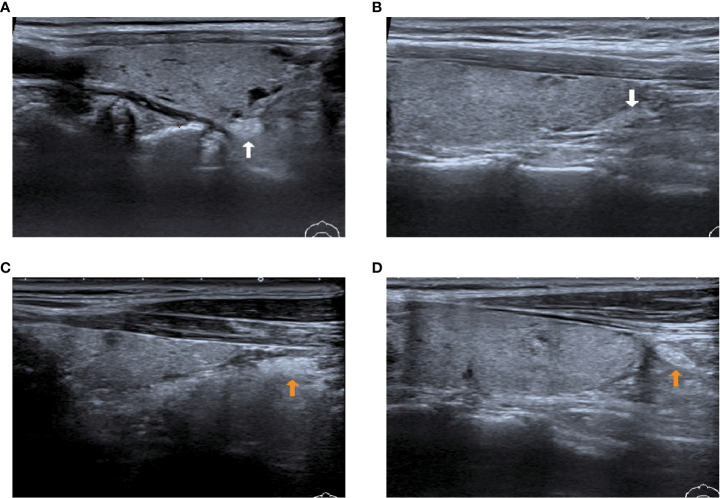
Two types of IPG observed by preoperative ultrasound images. **(A, B)**: White arrow shows type I PG, where the PGs are in close contact with the thyroid gland; **(C, D)**: Yellow arrow shows type II PG, where the PGs are free of the thyroid gland and not in close contact with the thyroid gland.

We have classified the position of IPGs into two categories (**Figure 6**): type I, where the IPGs were in close proximity to the thyroid gland and adhered to the thyroid peritoneum, on the inferior thyroid position or to the dorsal lateral thyroid peritoneum; type II, where the IPGs were not adhered to the thyroid peritoneum and separated from the thyroid gland for a certain distance.

### 3.3 Intraoperative comparison for IPG

After carefully searching the IPGs according to the preoperative ultrasound position, the coordinate parameters of parathyroid (A and B) were measured with a sterile scale, which were (33.70 ± 12.75) mm and (14.79 ± 8.82) mm, respectively, we found a good correlation (r= 0.8945; r= 0.9113) with the preoperative ultrasound measured spatial position (**Figure 5**). This indicates that our preoperative ultrasound-measured three-dimensional spatial coordinate was very helpful in finding identification during surgery.

Bilateral IPGs could be explored intraoperatively in 62 cases (73.8%) and at least one IPG was found in 74 cases (88.1%) in the preoperative ultrasound (**Table 2**). The percentage of those who could be found on preoperative ultrasonography but cannot be found intraoperatively, whether left or right sides, was less than 10% (6.0%, 1.2%). This indicates t that our preoperative ultrasonic identification technique has more than 90% (94%, 98.8%) accuracy (true positive rate) in identifying the tissue as a PG. Those who were not identified by preoperative ultrasound evaluation but had found intraoperatively were: 17 cases (20.2%) in the right side and 23 cases (27.4%) in the left side (false negative rate).

In addition, in the preoperative ultrasound group versus the control group, 74 (88.1%) vs. 60 (74.1%) IPGs could be detected intraoperatively on at least one side; 10 (11.9%) vs. 21 (25.9%) IPGs were not detected intraoperatively on both sides, P=0.0279 (**Table 3**).

**Table 3:** Comparisons of intra-operative localization of IPG in PUS Group and Control Group.

Patient numbers (n, %)	PUS Group (n=84)	Control Group (n=81)	P value
At least one IPG detected	74 (88.1%)	60 (74.1%)	0.0279 *
No IPG detected	10 (11.9%)	21 (25.9%)	

* Chi-square test.

We documented only the number of IPG detected intraoperatively in Control Group.

We can conclude that ultrasonic localization of the PGs can help in the search, identification and *in situ* protection of the PGs during thyroidectomy.

### 3.4 Evaluation of IPG protection after surgery

At first day postoperative, patients in the preoperative ultrasound group had better postoperative biochemical parameters including Ca^2+^: 2.12 (0.17) vs. 2.05 (0.31), P=0.03; PTH: 27.48 (14.88) vs. 23.27 (16.58), P=0.005, than the control group (**Table 4**). The risk of at least one type of reduced parathyroid function postoperatively: 26 (31.0%) vs. 41 (50.6%), P= 0.016.

**Table 4:** Ca2+, PTH levels and risks of hypoparathyroidism in PUS Group and Control Group.

	PUS Group(n=84)	Control Group(n=81)	P value
Ca2+ [mmol/L, median(quartiles)]			
pre-operatively	2.34 (0.10)	2.36 (0.17)	0.531 †
post-operative day 1	2.12 (0.17)	2.05 (0.31)	0.03 †
PTH [pg/ml, median(quartiles)]			
pre-operatively	43.38 (17.47)	41.02 (20.85)	0.234 †
post-operative day 1	27.48 (14.88)	23.27 (16.58)	0.005 †
Risk of hypoparathyroidism			
No risk (n, %)	58 (69.0%)	40 (49.4%)	0.016 *
At least one risk appeared (n, %)	26 (31.0%)	41 (50.6%)	
(i) Ca^2+^ less than 2.0 mmol/L (n)	15	32	–
(ii) PTH less than 15 pg/ml (n)	10	22	–
(iii) Numbness of mouth and lips (n)	13	17	–
(iv) Tingling and numbness of fingertips (n)	6	15	–
(v) Muscle aches and spasms (n)	1	5	–
(vi) Tetany (n)	0	0	–
(vii) Laryngeal and diaphragm spasm (n)	0	0	–

† Mann-Whitney U test.

* Chi-square test.

In addition, only 6 patients in the preoperative ultrasound group complained of slight numbness in both hands-on postoperative day 1, and only 1 complained of numbness and tingling with spasm in the fingertips; none of them complained of the above-mentioned numbness on postoperative day 3 when they were left the hospital. In contrast, 15 patients in the control group complained of slight numbness in both hands first day after surgery, and 5 patients complained of numbness and tingling in the with spasm; 2 patients complained of numbness in both hands and around the mouth again on the third day after surgery. There were no critical symptoms such as hand-foot convulsions and laryngeal diaphragm spasms in the study and control groups after surgery.

## 4 Discussion

In present study, we evaluated the effect of the application of preoperative ultrasound localization techniques on reducing the risk of postoperative hypoparathyroidism. We found that our preoperative ultrasonic identification technique has more than 90% (94%, 98.8%) accuracy (true positive rate) in identifying the tissue as a PG. Postoperative biochemical result showed a better Ca^2+^ and PTH levels at first day post-operation in PUS group compared to control group.

**Figure 7:**
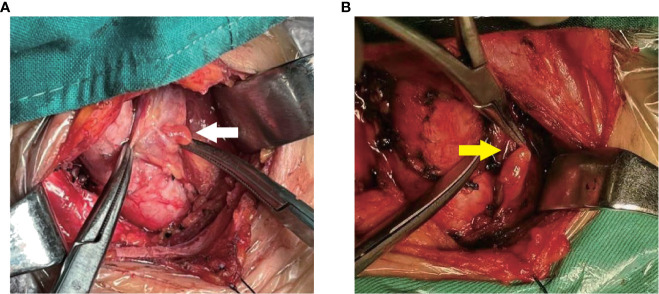
Two types of IPG observed during surgery. **(A)**: White arrow shows Type I PG; **(B)**: yellow arrow shows Type II PG.

Interestingly, As shown in **Table 5**, we found a higher detection rate of type II PG than type I pg. We suppose the reasons are as follows: Firstly, type II IPG are not closely adhered to thyroid, which makes them easier to identify; Secondly, most type II IPG were enveloped by a “fat capsule” (**Figure 7**).

**Table 5:** IPG location types and detection rates.

Patient numbers (n, %)	Statistics
Bilateral IPG Type I detected	11 (13.1%)
Right IPG Type I detected both pre- and intra-operatively	10 (11.9%)
Left IPG Type I detected both pre- and intra-operatively	9 (10.7%)
Bilateral IPG Type I detected both pre- and intra-operatively	3 (3.6%)
Bilateral IPG Type II detected	18 (21.4%)
Right IPG Type II detected both pre- and intra-operatively	35 (41.7%)
Left IPG Type II detected both pre- and intra-operatively	37 (44.0%)
Bilateral IPG Type II detected both pre- and intra-operatively	14 (16.7%)
No IPG detected both pre- and intra-operatively	6 (7.1%)

Type I: Closely opposed to the thyroid peritoneum; Type II: Free below the inferior pole of the thyroid or lateral to the thyroid, not closely opposed to the thyroid.

The reasons for the high false-negative rate are as follows: (1) A small percentage of suspicious IPGs on ultrasound do not have clear borders and echogenicity and are similar to surrounding fat, lymph, and connective tissue. The three senior ultrasound specialists in our team did not include ambiguous tissues in the positive criteria. (2) A small percentage of IPGs do not visible clearly on ultrasound and may be covered by other tissues. For example, they are extremely close to the true peritoneum of the thyroid gland, far from the thyroid gland, or encapsulated in lymph nodes in the central region and not clearly detected.

At present, the SPGs are very challenging to visualize on ultrasonography, the result as follows: (1) We have tried to obtain satisfactory ultrasound images for the SPGs from experienced ultrasonographers. Very few of the SPGs are clearly visualized on ultrasound. This may be due to the complex anatomy surrounding the SPGs, such as the thyroid cartilage, which affects the visualization on ultrasound. (2) The location of the SPGs is relatively fixed and without surrounding fat or lymph nodes. As a result, the SPGs are easy to find during surgery. (3) The location of the IPGs is extremely variable, with a complex blood supply and surrounding fat or lymph nodes. During the operation, it is difficult to find, which increased the risk of accidental removal or blood supply damage. (4) In our prior experience, the probability of accidental removal of the IPGs is much higher than the SPGs. Therefore, our study focused on bilateral inferior parathyroid glands and excluded bilateral superior parathyroid glands.

How it helps to reduce the damage to the parathyroid glands and their blood supply by anticipation the location of the inferior parathyroid glands? Since we anticipate their location, we can protect the parathyroid glands and their blood supply before they are fully exposed. Our technique reduces the window period to discover the parathyroid glands. More importantly, our technique avoids unnecessary manipulations to search and expose parathyroid (this is also the main reason of accidently damage by experienced surgeon), that effectively reduces the risk of the direct damage of the parathyroid glands. Because while we were searching for it during surgery, we had already damaged it or its blood supply.

For the parathyroid glands that closely attached to thyroid (type I IPG), we can try to anticipately preserve their blood supply before thyroidectomy. For the parathyroid glands that positioned at a certain distance from the thyroid gland (type II IPG), due to their high similarity to surrounding lymph nodes and fatty granules, surgeon should well expose the suspicious structures one by one to identify the parathyroid. Our preoperative localization technique allows us to anticipate its location and avoid exposing every suspicious structure, which reduces the risk of accidental removal or blood supply damage.

Additionally, for the parathyroid glands that closely attached to thyroid glands and without independent blood supply. This type of parathyroid glands could not be preserved in situ, and the only solution is auto-transplantation. We have excluded these cases in the present works. This is because survival rates are different after auto-transplantation. Serum Ca2+ and PTH may be affected.

In our study, in 10 cases (11.9%) we did not find IPG either by preoperative ultrasound localization or intraoperatively. The IPG and SPG have distinct embryonal origin: The IPG originates from the third pharyngeal bursa together with the thymus, whereas the SPG originates from the fourth pharyngeal bursa (20, 21), which explains the variation in the location of the IPGs. The 10 cases in which we did not find the IPGs could localized around the vascular sheaths of the neck, in the thymus, mediastinum or thyroid parenchyma. This category may be called “type III” PG.

Currently, 99mTc-MIBI is only used to localize parathyroid adenomas but is not recommended for the identification of normal PGs during surgery (22, 23). Although exogenous dyes such as indocyanine green have been used intraoperatively to localize PGs, it may cause toxicity, pain, allergy (24). In addition, there is the parathyroid near-infrared autofluorescence technique. However, it is difficult to determine type II PG, which is covered by fat, connective tissue or the thyroid gland. In other word, the type II PGs cannot be identified with autofluorescence unless the glands are exposed (25, 26). More importantly, these techniques require additional manipulations and increase overall time cost of surgery.

## 5 Conclusion

In summary, our ultrasonography identification and localization help to anticipate the location of parathyroid gland before surgery. Our technique reduces intraoperative damage to the parathyroid glands by reducing unnecessary manipulations during the search of the parathyroid glands, which reduces the incidence of postoperative hypoparathyroidism. We have confirmed its effect by postoperative biochemical analysis. In addition, this technique also reduces the psychological stress of the surgeon during surgery about the unknown location of the parathyroid glands.

## Data availability statement

The original contributions presented in the study are included in the article/supplementary material. Further inquiries can be directed to the corresponding author.

## Ethics statement

The studies involving human participants were reviewed and approved by the ethics committee of Shandong Provincial Hospital Affiliated to Shandong First Medical University. Written informed consent for participation was not required for this study in accordance with the national legislation and the institutional requirements.

## Author contributions

CG designed and developed whole project and modified original article. RC, KZ reviewed and editing preparation, creation and presentation of the work of the research group. RC written the original article and completed the statistical work. JL, LG, and KL assisted measurement of IPGs in surgery. All authors contributed to the article and approved the submitted version.
